# Neuronal impairment following chronic *Toxoplasma gondii* infection is aggravated by intestinal nematode challenge in an IFN-γ-dependent manner

**DOI:** 10.1186/s12974-019-1539-8

**Published:** 2019-07-29

**Authors:** Timothy French, Henning Peter Düsedau, Johannes Steffen, Aindrila Biswas, Norus Ahmed, Susanne Hartmann, Thomas Schüler, Björn H. Schott, Ildiko Rita Dunay

**Affiliations:** 1 Institute of Inflammation and Neurodegeneration, Medizinische Fakultät, Otto-von-Guericke-University Magdeburg, Leipziger Straße 44, 39120 Magdeburg, Germany; 2 Institute of Molecular and Clinical Immunology, Otto-von-Guericke-University Magdeburg, Magdeburg, Germany; 3 Department of Veterinary Medicine, Institute of Immunology, Free University Berlin, Berlin, Germany; 4 Leibniz Institute of Neurobiology, Magdeburg, Germany; 5 Center for Behavioral Brain Sciences, Magdeburg, Germany; 6 Department of Psychiatry and Psychotherapy, University Medicine Göttingen, Göttingen, Germany

**Keywords:** *Toxoplasma gondii*, *Heligmosomoides polygyrus*, Co-infection, Immunomodulation, Neuroinflammation

## Abstract

**Background:**

It has become increasingly evident that the immune and nervous systems are closely intertwined, relying on one another during regular homeostatic conditions. Prolonged states of imbalance between neural and immune homeostasis, such as chronic neuroinflammation, are associated with a higher risk for neural damage. *Toxoplasma gondii* is a highly successful neurotropic parasite causing persistent subclinical neuroinflammation, which is associated with psychiatric and neurodegenerative disorders. Little is known, however, by what means neuroinflammation and the associated neural impairment can be modulated by peripheral inflammatory processes.

**Methods:**

Expression of immune and synapse-associated genes was assessed via quantitative real-time PCR to investigate how *T. gondii* infection-induced chronic neuroinflammation and associated neuronal alterations can be reshaped by a subsequent acute intestinal nematode co-infection. Immune cell subsets were characterized via flow cytometry in the brain of infected mice. Sulfadiazine and interferon-γ-neutralizing antibody were applied to subdue neuroinflammation.

**Results:**

Neuroinflammation induced by *T. gondii* infection of mice was associated with increased microglia activation, recruitment of immune cells into the brain exhibiting Th1 effector functions, and enhanced production of Th1 and pro-inflammatory molecules (IFN-γ, iNOS, IL-12, TNF, IL-6, and IL-1β) following co-infection with *Heligmosomoides polygyrus*. The accelerated cerebral Th1 immune response resulted in enhanced *T. gondii* removal but exacerbated the inflammation-related decrease of synapse-associated gene expression. Synaptic proteins EAAT2 and GABA_A_α1, which are involved in the excitation/inhibition balance in the CNS, were affected in particular. These synaptic alterations were partially recovered by reducing neuroinflammation indirectly via antiparasitic treatment and especially by application of IFN-γ-neutralizing antibody. Impaired iNOS expression following IFN-γ neutralization directly affected EAAT2 and GABA_A_α1 signaling, thus contributing to the microglial regulation of neurons. Besides, reduced CD36, TREM2, and C1qa gene expression points toward inflammation induced synaptic pruning as a fundamental mechanism.

**Conclusion:**

Our results suggest that neuroimmune responses following chronic *T. gondii* infection can be modulated by acute enteric nematode co-infection. While consecutive co-infection promotes parasite elimination in the CNS, it also adversely affects gene expression of synaptic proteins, via an IFN-γ-dependent manner.

**Electronic supplementary material:**

The online version of this article (10.1186/s12974-019-1539-8) contains supplementary material, which is available to authorized users.

## Background

Immune activity in the central nervous system (CNS) historically has been thought to almost invariably reflect pathological processes [1–3]. However, inflammation is an integral component of neurobiological processes supporting tissue repair, synaptic pruning, pathogen removal, and inflammation resolution to maintain homeostasis [4]. Still, there is the physical segregation of the nervous and immune systems via the blood-brain barrier (BBB), separating the CNS from the peripheral cytokine milieu and allows for more controlled neuro-immune activity. However, when the BBB is compromised, as in neuroinflammation, the brain parenchyma becomes threatened as there is an increased activation of both resident and recruited immune activity [5]. Interferon-γ (IFN-γ), a crucial type 1 cytokine, for example, has been shown to modulate the activity of the principal inhibitory neurotransmitter gamma-aminobutyric acid (GABA), thereby affecting neuronal connectivity and social behavior [6]. Pro-inflammatory cytokines such as tumor necrosis factor (TNF), interleukin (IL)-6, or IL-1β have been linked to neuronal cell death via both apoptosis and necrosis and to cognitive and motor dysfunction [7, 8]. Furthermore, downregulation of pro-inflammatory myeloid cell activity via the type 2 cytokine IL-4 produced by T cells has been associated with increased release of brain-derived neurotrophic factor (BDNF) and reversal of memory impairment [9]. Conversely, microglia, the resident innate immune cells of the CNS, have been shown to express receptors for multiple neurotransmitters including GABA, glutamate, or dopamine [10–13]. These findings highlight a rather pleiotropic role of the respective proteins in normal physiology, with their functions depending on their location. In chronic neuroinflammation, there is a prolonged activated and recruited immune presence in the CNS and neural and immune functions can become dysbalanced, increasing the risk for secondary neural damage. This is evident in the strong association of chronic neuroinflammation with neurodegenerative diseases such as Alzheimer’s disease (AD) [14], Parkinson’s disease (PD) [15], amyotropic lateral sclerosis (ALS) [16], and the profound inflammation-related neural damage found in multiple sclerosis (MS) [17].

Invasion of the CNS by neurotropic pathogens is a common cause of chronic neuroinflammation [18]. *Toxoplasma gondii* (*T. gondii*) preferentially infects neurons where it forms cysts that remain for the lifetime of the host causing persistent subclinical neuroinflammation [19, 20]. The conversion of fast-replicating tachyzoites to slow-replicating bradyzoites and the subsequent formation of a cyst wall are induced by the host’s adaptive immune response. T cells are critical in preventing the reactivation of bradyzoite-filled cysts, as evident in the prolonged low-grade cerebral inflammation to control chronic infection seen in immunocompetent hosts [21, 22]. Neuroinflammation is initially facilitated when resident microglia and astrocytes first encounter the invading parasites, which leads to the release of chemokines and pro-inflammatory mediators (i.e., TNF, IL-6, or IL-1β) followed by recruitment of peripheral myeloid and lymphoid cells [23–25]. Myeloid cells support chronic neuroinflammation by their capacity to produce pro-inflammatory cytokines such as IL-12, which is crucial in maintaining the production of IFN-γ. Produced primarily by CD4^+^ and CD8^+^ T cells as well as innate lymphoid cells [26, 27], IFN-γ is an essential factor in host protection against the parasite, as it triggers multiple immune cell mechanisms leading to the elimination of the parasite [28]. Moreover, IFN-γ can activate the choroid plexus endothelium, thereby aiding the migration of leukocytes into the CNS parenchyma [29, 30].

*T. gondii* infection has been shown to alter behavior in rodents [29], and in humans, seropositive individuals have been shown to exhibit subclinical behavioral alterations as well as an increased risk for neuropsychiatric disorders [30] such as PD [31], AD [32], or schizophrenia [33]. Conclusively, a recent large-scale study provided compelling evidence for the association between latent *T. gondii* infection and schizophrenia in humans [18]. Distinctly, the immune response to *T. gondii* and the neuropathological mechanisms of these disorders overlap by involving the same cell types and, at least in part, the same molecules. We have previously demonstrated that chronic *T. gondii* infection leads to a loss of structural complexity of axons and dendrites, primarily in the neocortex and hippocampus [34]. Moreover, we have recently shown that the synaptic protein composition was altered, in particular, with a downregulation of components of glutamatergic signaling as a consequence of the inflammatory milieu induced by chronic infection [35].

A common clinical approach for treating neuroinflammatory disorders has been the application of immunosuppressive or anti-inflammatory drugs, to slow down disease progression, but side effects are often severe [36, 37]. In the case of infections underlying neuroinflammation, as in cerebral toxoplasmosis, this is particularly dangerous as immunocompromised individuals are at risk for reactivation of life-threatening acute Toxoplasma encephalitis (TE) [38, 39]. More recently, immunomodulatory approaches are being applied to modify the immune response without removing the homeostatic functions of cells such as tissue maintenance and repair [40–42]. Helminths administered as immunotherapy have gained significant attention, where live parasitic worms or worm-derived molecules are applied as treatment of chronic inflammatory diseases [43–45]. By taking advantage of helminths’ natural abilities to create an anti-inflammatory environment, helminth infection and helminth-derived molecules have the potential to beneficially modulate immune responses to potentially pre-existing diseases. Helminth immunomodulation has already shown success in human models of inflammatory bowel diseases [46, 47] and multiple sclerosis (MS) [48–50] as well as murine models of allergic airway hyperreactivity [51–53] rheumatoid arthritis (RA), type 1 diabetes, and experimental autoimmune encephalomyelitis (EAE) [44, 54, 55]. Helminths primarily achieve this immunomodulation via induction of regulatory circuits. In general, helminth infections stimulate the release of IL-4, IL-5, IL-10, and IL-13, which shifts T cell responses towards a Th2 response [56]. Regulatory T cells (Tregs) as well as regulatory macrophages are also stimulated, leading to increased production of anti-inflammatory cytokines IL-10 and transforming growth factor beta (TGF-β), which corroborate the helminths’ induced anti-inflammatory environment [57]. Scientific interest continues to grow around helminth-dependent immune modulation, especially in response to the increasing incidence of allergies and auto-immune diseases [58, 59]. However, there is insufficient knowledge about the potential effects of peripheral helminth infections on concurrent inflammatory processes in the CNS.

In the present study, we aimed to elucidate how immunological and neural sequelae of *T. gondii*-induced neuroinflammation are altered by a concomitant intestinal helminth infection and resulting immunomodulation. To this end, we administered the strictly enteric intestinal nematode *Heligmosomoides polygyrus* (*H. polygyrus*) to mice previously infected with *T. gondii. H. polygyrus* infection is known to lead to a strong induction and systemic dissemination of Th2-type responses during the acute phase of infection [60, 61]. Our data revealed that a co-infection of *T. gondii*-infected mice with *H. polygyrus* induced an increase of the recruitment of Ly6C^hi^ inflammatory monocytes and subsequent increase in the Th1 immune response of resident and recruited immune cells, characterized by IFN-γ, IL-12, iNOS, and MHC II expression. This upregulation of the Th1 response and inflammatory cell recruitment ultimately led to a significant reduction of the parasite burden in co-infected mice. However, this was accompanied by a reduction in neural markers, as determined by synaptic gene expression changes. Notably, the synaptic alterations were partially recovered by limiting infection, and thus neuroinflammation with sulfadiazine, or by application of IFN-γ-neutralizing antibodies. IFN-γ-dependent synaptic pruning might be one possible explanation for the observed neuronal changes.

## Methods

### Animals

Experiments were conducted using female, wild type (WT) C57BL/6 mice (8 weeks old; purchased from Janvier Laboratories, Cedex, France) bred under specific pathogen-free (SPF) conditions. All mice were group-housed in 12-h day/night cycles at 22 °C with free access to food and water. All animal experiments were approved by local authorities according to German and European legislation.

### *Toxoplasma gondii* infection

*T. gondii* cysts of type II strain ME49 were harvested from the brains of female NMRI mice infected with *T. gondii* cysts 8–12 months earlier, as described previously [34]. In short, isolated brains were mechanically homogenized in 1-ml sterile phosphate-buffered saline (PBS), and the number of cysts in the homogenate was determined using a light microscope. Mice were infected with two cysts via oral gavage (concentration adjusted to 2 cysts per 200 μl).

### *Heligmosomoides polygyrus* infection

*H. polygyrus* was retained by serial passage in C57BL/6Rj mice as previously described [62]. Mice were infected by oral gavage with 200 L3 larvae in 200 μl PBS.

### Worm fecundity and worm burden

Adult worms were isolated from the small intestine and counted. Female worms were subsequently kept individually (8 per mouse) in a 96-well round-bottom plate containing RPMI, 200 U/ml penicillin, and 200 μg/ml streptomycin (all from PAA, Austria) at 37 °C. After 24 h, female *H. polygyrus* adults were removed from the wells and fecundity was determined by counting the eggs shed per female worm using a binocular microscope.

### Sulfadiazine treatment

A solution of 400 mg/L of sulfadiazine in drinking water was prepared and then autoclaved [63]. Mice received sulfadiazine treatment in their drinking water ad libitum. Treatment began on day 10 post-infection and continued until sacrifice on day 28 post-infection.

### IFN-γ neutralization

IFN-γ was neutralized by administration of monoclonal antibody against IFN-γ (clone: XMG1.2, DRFZ, Berlin). Mice were treated intraperitoneally (i.p.) with 0.5 mg anti-IFN-γ in 200 μl PBS on days 14, 17, 20, 23, and 26 post-infection [64]. Isotype IgG1 antibody (clone: 19e1, DRFZ, Berlin) was used as control. Saline injections were administered to non-infected and *H. polygyrus*-only-infected mice to control for the antibody treatment.

### Organ collection

First, mice were deeply anesthetized by isoflurane inhalation (Baxter). Subsequently, mice were transcardially perfused with sterile ice-cold PBS. Brains were removed and stored in RPMI medium (Life Technologies) or RNA*later* (Qiagen) for additional analysis. Samples were stored in RNA*later* (Qiagen), were kept at 4 °C overnight, and then transferred to − 20 °C. Samples in RPMI medium were stored on ice until further experimental procedures.

### Cell isolation

To isolate brain immune cells, brains were homogenized in a buffer containing 1 M HEPES (pH 7.3) and 45% glucose and then filtered through a 70-μm strainer. Leukocytes were separated via Percoll gradient centrifugation (GE Healthcare) as described previously [65].

### Flow cytometric analysis

Single-cell suspensions were incubated with an anti-FcγIII/II receptor antibody (clone 93, eBioscience) to block unspecific binding and Zombie NIR™ (Biolegend), a fixable viability dye. Thereafter, cells were stained with fluorochrome-conjugated antibodies against cell surface markers: CD45 (30-F11), CD11b (M1/70), Ly6C (HK1.4), MHCI (28-14-8), and MHCII (M5/114.15.2) all purchased from eBioscience; CD3 (17A2), CD4 (RM4-5), and CD8α (53-6.7) all purchased from Biolegend; and Ly6G (1A8) purchased from BD Biosciences in FACS buffer at 4 °C for 30 min and then fixed in 4% paraformaldehyde (PFA, Affymetrix). Matched FMO controls were used to assess the level of background fluorescence in the respective detection channel.

Intracellular staining was performed as previously described [66]. Afterwards, cells were incubated with anti-FcγIII/II receptor antibody (clone 93, eBioscience) and Zombie NIR™ (Biolegend). Surface epitopes were then stained with CD45 (30-F11), CD11b (M1/70), Ly6C (HK1.4), Ly6G (1A8), CD3 (17A2), CD4 (RM4-5), and CD8α (53-6.7). To measure cytokine expression, cells were stained with TNF (MP6-XT22), IL-12p40 (C17.8), IL-10 (JES5-16ES), purchased from eBioscience; iNOS (6, BD Biosciences); and IFN-γ (XMG1.2, Biolegend). Matched isotype controls were used to assess the level of non-specific binding.

Flow cytometric analysis was performed on BD FACS CANTO II (BD Bioscience) and on Attune NxT Flow Cytometer (Thermo Fisher) and analyzed with FlowJo (version 10, Tree star).

### DNA and RNA isolation

Samples stored in RNA*later* were homogenized in BashingBeads tubes (Zymo Research, Freiburg, Germany). AllPrep DNA/RNA Mini Kit (Qiagen) was used to isolate DNA, and the peqGOLD total RNA kit (Peqlab, Erlangen, Germany) was used to isolate total RNA from the homogenate following the manufacturer’s instructions, respectively.

### Semiquantitative PCR and RT-qPCR

*T. gondii* burden was determined using the FastStart Essential DNA Green Master kit (Roche). The target *T. gondii* gene used was *TgB1*, and *Mm. Asl* (TIBMolbiol, Berlin, Germany) was used as a reference. The stage of parasite burden was quantified using the Power SYBR® Green RNA-to-CT™ 1-Step Kit (Thermo Fisher) for bradyzoite-specific Bag1 and tachyzoite-specific Sag1 using Gapdh as reference gene. All genes were purchased from TIBMolbiol, Berlin, Germany.

Relative gene expression was determined similar to previous descriptions [67, 68] using the TaqMan® RNA-to-CT™ 1-Step Kit (Life Technologies). TaqMan® Gene Expression Assays (Life Technologies) were used for mRNA amplification of *Ifn-γ* (Mm00801778_m1), *Il10* (Mm00439616_m1), *Tnf* (Mm00443258_m1), *Il1b* (Mm00434228_m1), *Il12a* (Mm00434165_m1), *Arg1* (Mm00475988_m1), *Il6* (Mm00446190_m1), *Il13* (Mm00434204_m1), *Il4* (Mm0445259_m1), *Nos2* (Mm00440485_m1), *Trem2* (Mm04209422_m1), *Bdnf* (Mm04230607_s1), *Syp* (Mm00436850_m1), *Dlg4* (Mm00492193_m1), *Slc1a2* (Mm01275814_m1), *Gabra1* (Mm00439046_m1), and *C1qa* (Mm00432142_m1). Expression of *Hprt* (Mm01545399_m1) was chosen as reference, and target/reference ratios were calculated with the LightCycler® 96 software version 1.1 (Roche). All results were further normalized to the mean of the corresponding non-infected control group.

### Statistical analysis

Data sets were statistically analyzed using SPSS 22.0 (IBM, Armonk, NY). To account for the factorial nature of our experiments and the multiple measures of outcome, we computed ANOVA models with infection status and or treatment as between-subjects factors and different outcome measures (e.g., cell counts, gene expression) as within-subject factors or dependent variables. Whenever we could assume a positive correlation between outcome measures, ANOVAs for repeated measures were employed, and otherwise, MANOVAs were used. When data from different series of experiments or different PCR plates were analyzed in the same statistical model, appropriate covariates were included. Whenever a within-subjects factor had more than two levels, degrees of freedom were corrected for non-sphericity using the Greenhouse-Geisser correction. ANOVAs with significant effects of interest were followed by planned *t* test comparisons to assess directionality of the observed effects. Owing to the small sample sizes, unequal variances were assumed in all *t* tests. The significance level was set to *p* < .05 for all statistical comparisons, and two-tailed *p* values are reported for all *t* tests unless otherwise stated.

Diagrams were generated using GraphPad Prism 6.0 (GraphPad Software). Data are presented as mean ± SEM.

## Results

### *H. polygyrus* co-infection amplifies the Th1 response to *T. gondii* in the brain

**Fig. 1:**
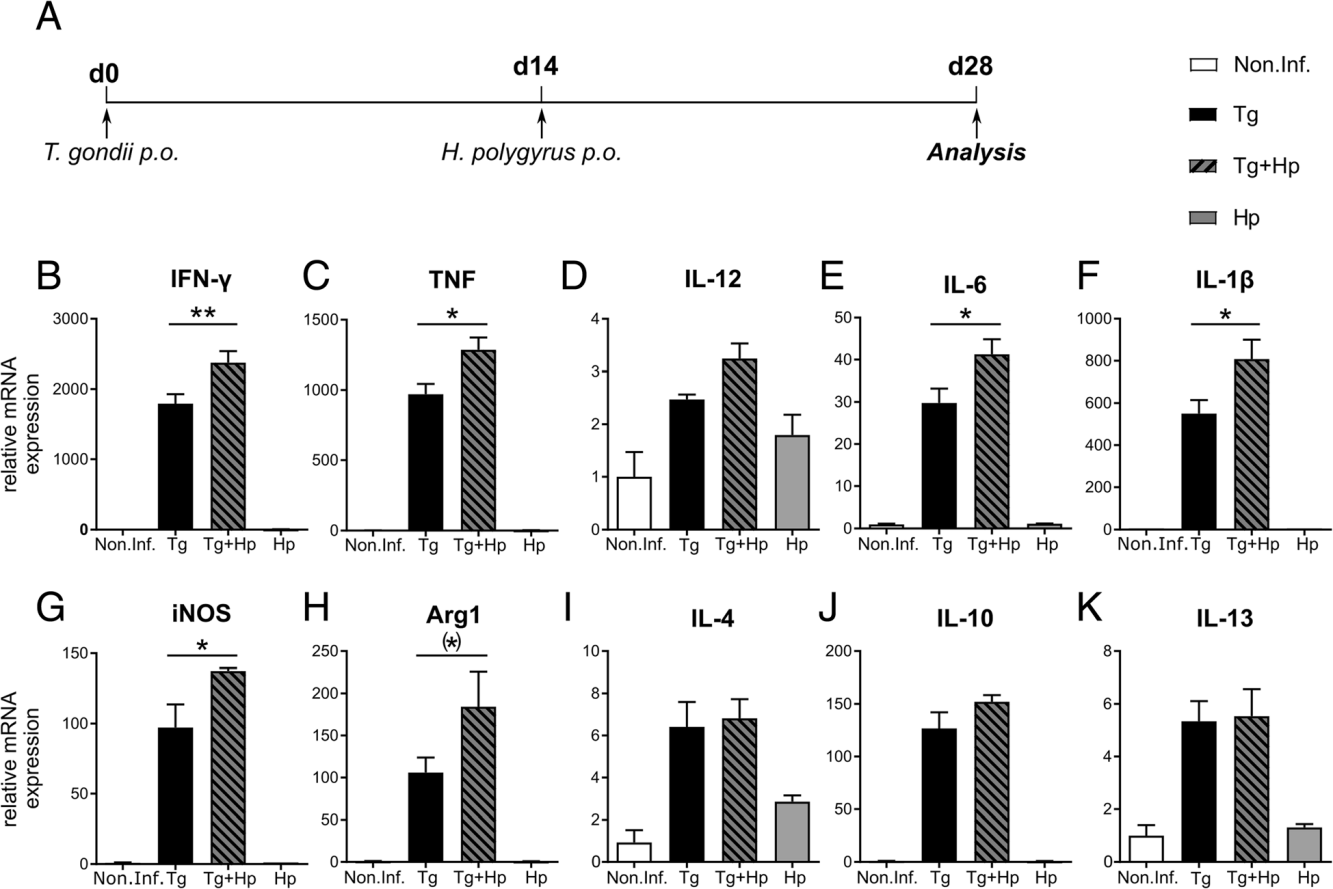
Type 1 immune response in the brain increased upon co-infection with *H. polygyrus.* **a** Experimental setup: C57BL/6 mice were first infected p.o. with *T. gondii* (2 cysts, ME49). On day 14 p.i., mice were infected p.o. with *H. polygyrus* (200 L3 larvae). **b**–**k** qRT-PCR data for relative expression of mRNA in whole brain homogenate from non-infected (Non.Inf.), *T. gondii*-infected (Tg), *T. gondii* and *H. polygyrus* co-infected (Tg+Hp), and *H. polygyrus*-infected (Hp) mice. Relative mRNA levels were normalized to the mean of the non-infected control group (Non.Inf). Bars represent mean ± SEM

On day 14 post *T. gondii* infection, after the parasites had crossed the blood-brain barrier (BBB) and provoked a CNS immune response, mice were co-infected with *H. polygyrus*. Immune cell collection took place on day 28, thus 14 days post *H. polygyrus* infection and during the acute phase of the helminth infection (Fig. 1a). To determine whether an acute intestinal helminth infection affects *T. gondii*-dependent neuroinflammation, we used qRT-PCR to examine changes in cytokine gene expression levels in whole-brain homogenates (Fig. 1b–k). Gene expression analysis of the pro-inflammatory cytokines IFN-γ, TNF, and IL-12, which have previously been implicated in the immune response to *T. gondii* [69, 70], showed significant effects of both *T. gondii* (*F*_1,14_ = 311.85, *p* < 0.001; three-way ANOVA for repeated measures with cytokine as within-subjects factor and *T. gondii* and *H. polygyrus* as between-subjects factors) and *H. polygyrus* (*F*_1, 14_ = 11.55, *p* = 0.004) infection, as well as an interaction of the two pathogens (*F*_1, 14_ = 10.77, *p* = 0.005). We further observed interactions of both pathogens and their combination with cytokine, reflecting differential effects on IFN-γ, TNF, and IL-12 (all *F* > 8.40, all *p* < 0.010). Specifically, co-infected mice compared to mice infected with *T. gondii* only showed significantly higher expression of IFN-γ (post hoc two-sample *t* test: *t*_7.4_ = 3.51, *p* = 0.009) and TNF (*t*_7.7_ = 2.74, *p* = 0.026), while the difference in IL-12 expression was not significant (*p* = 0.591) (Fig. 1b–d). *H. polygyrus*-infected mice compared to uninfected mice, on the other hand, exhibited significantly increased expression of TNF (*t*_6.9_ = 2.82, *p* = 0.026), but not IFN-γ or IL-12 (all *p* > 0.238).

When testing for expression changes of IL-4, IL-10, and IL-13 as mediators of the Th2 or anti-inflammatory responses, we found a significant main effect of *T. gondii* infection (*F*_1, 12_ = 206.22, *p* < 0.001; three-way ANOVA for repeated measures with cytokine as within-subjects factor and *T. gondii* and *H. polygyrus* as between-subjects factors) as well as a cytokine by *T. gondii* interaction (*F*_1.0, 12.2_ = 147.70, *p* < 0.001), but no effects related to *H. polygyrus* infection alone or in interaction with *T. gondii* interaction (all *p* > 0.187) (Fig. 1e–g). Together with the additive effects of *T. gondii* and *H. polygyrus* on IFN-γ and TNF expression, these results suggest that a co-infection with the enteric nematode *H. polygyrus* during the acute phase of the helminth infection shifts the balance of Th1/Th2 responses towards an increased Th1-type cytokine response.

In an exploratory analysis, we further assessed the effects of *T. gondii* and *H. polygyrus* infection on several additional markers previously implicated in the immune responses to *T. gondii* infection (Arg1, IL-6, IL-1β, and iNOS; Fig. 1h–k; see [71]), revealing effects of both *T. gondii* (three-way ANOVA for repeated measures with gene as within-subjects factor and *T. gondii* and *H. polygyrus* infection as between-subjects factors: *F*_1, 12_ = 145.05, *p* < 0.001) and *H. polygyrus* (*F*_1, 12_ = 7.10, *p* = 0.021) as well as an interaction (*F*_1, 12_ = 7.13, *p* = 0.020). The observed interaction was driven by increased expression of IL-1β (*t*_5.9_ = 2.29, *p* = 0.033, one-tailed), IL-6 (*t*_6.8_ = 2.33, *p* = 0.027, one-tailed), and iNOS (*t*_4.1_ = 2.41, *p* = 0.036, one-tailed) in co-infected animals compared to animals infected with *T. gondii* only, whereas no single marker showed a significant difference between uninfected mice and mice infected with *H. polygyrus* only (all one-tailed *p* > 0.155).

Given the more pronounced clearance of *T. gondii* and lack of a Th2 response in co-infected mice, we investigated the immune response to *H. polygyrus*. To this end, we examined the gene expression of Th2 cytokines IL-4 and IL-13 (characteristic of *H. polygyrus* infection) in the spleens of mice (Additional file 1: Figure S1A-B). There was a pronounced interactive effect of the parasites on cytokine infection (*F*_1, 14_ = 308.30, *p* < 0.001; three-way ANOVA for repeated measures with cytokine as within-subjects factor and *T. gondii* and *H. polygyrus* as between-subjects factors), reflecting the significantly higher expression of both IL-4 and IL-13 in mice infected with *H. polygyrus* only compared to all other groups (all *t* > 11.93, all *p* < 0.001). Furthermore, both mice infected with *T. gondii* alone and co-infected animals showed lower splenic expression of IL-4 and IL-13 compared to uninfected mice (all *t* > 8.76, all *p* < 0.002). From these data, we concluded that mice were capable of manifesting an immune response to *H. polygyrus*, but that their immune response was shifted from a Th2- to Th1-type response when *T. gondii* infection was present.

### Altered immune response results in reduced parasite burden in the brain

**Fig. 2:**
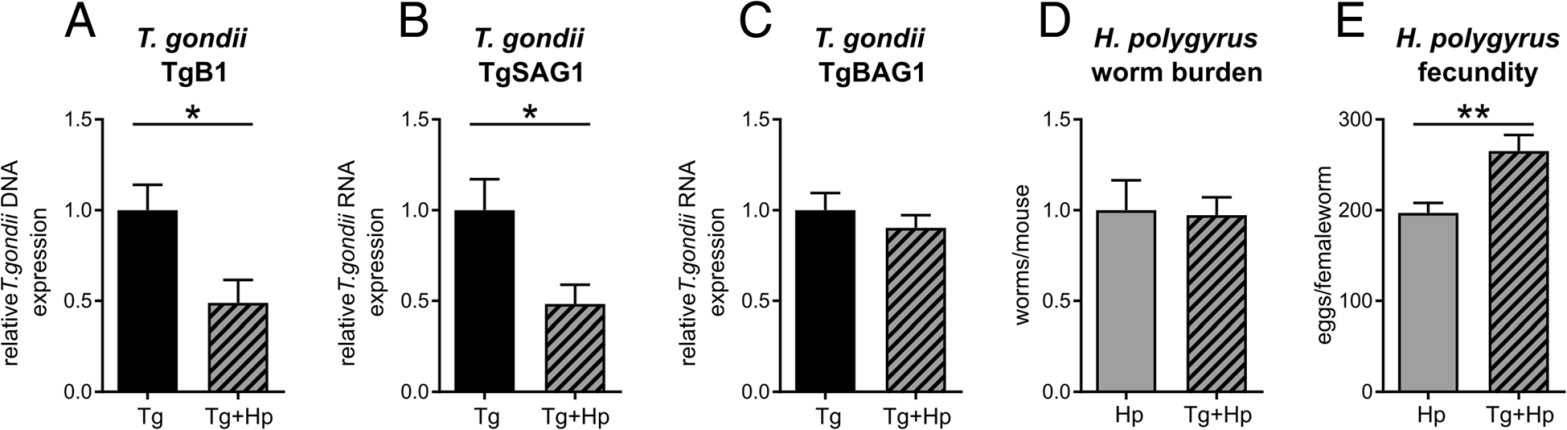
Parasite burden decreases and worm fecundity increases upon *H. polygyrus* co-infection. **a**–**c** *T. gondii* parasite burden in the brains of *T. gondii* infected (Tg) and *T. gondii* and *H. polygyrus* co-infected (Tg+Hp) mice. qPCR and qRT-PCR data of relative expression of *T. gondii* genes *TgB1* (**a**), Sag1 (**b**), and Bag1 (**c**), normalized to the *T. gondii* infected (Tg) group. **d** *H. polygyrus* worm burden. **e** Fecundity of female worms. Bars represent mean ± SEM

To investigate if the immune response reflected by the increase in *T. gondii*-related cytokines influenced the overall parasite burden, we measured DNA levels of *T. gondii*-specific gene *TgB1* in the brains of infected animals. TgB1 DNA levels were significantly decreased in co-infected animals compared to mice infected with *T. gondii* only (*t*_7.9_ = 3.24, *p* = 0.012; Fig. 2a). Expression analysis of stage-specific genes for tachyzoites (*Tgsag1*) and bradyzoites (*Tgbag1*) further showed that the decreased parasite burden was largely attributable to fast-replicating, highly virulent tachyzoites (*t*_7.5_ = 2.86, *p* = 0.022; Fig. 2b), while the burden of less virulent, slow-replicating bradyzoites did not change significantly (*t*_7.4_ = 0.83, *p* = 0.433; Fig. 2c). These results suggest that the increased Th1 response in co-infected mice actually increased the effectiveness of *T. gondii* elimination.

In accordance with these results, we next investigated how the co-infection affected the ability to control the *H. polygyrus* infection. To this end, we examined worm burden and worm fecundity from co-infected compared to *H. polygyrus*-only infected animals (Fig. 2d–e). We observed no significant change in worm burden (*F*_1, 20_ = 0.02, *p* = 0.889; ANCOVA with infection status as fixed factor and experiment as covariate). Regarding worm fecundity in co-infected animals compared to mice infected with *H. polygyrus* alone, we observed nominally lower egg counts in co-infected animals, although this did not reach significance when testing conservatively (ANCOVA with average egg count per mouse as dependent variable, infection status as fixed factor, and standard deviation of egg count and number of worms counted as covariates: *F*_1, 6_ = 2.46, *p* = 0.168). When directly comparing the number of eggs per worm as a function of infection status, the average number of eggs was indeed significantly higher in co-infected mice (*t*_60.3_ = 3.28, *p* = 0.002), suggesting a (minor) detrimental effect of *T. gondii* infection on helminth elimination.

### Acute *H. polygyrus* co-infection amplifies Th1 response of brain recruited and resident immune cells

**Fig. 3:**
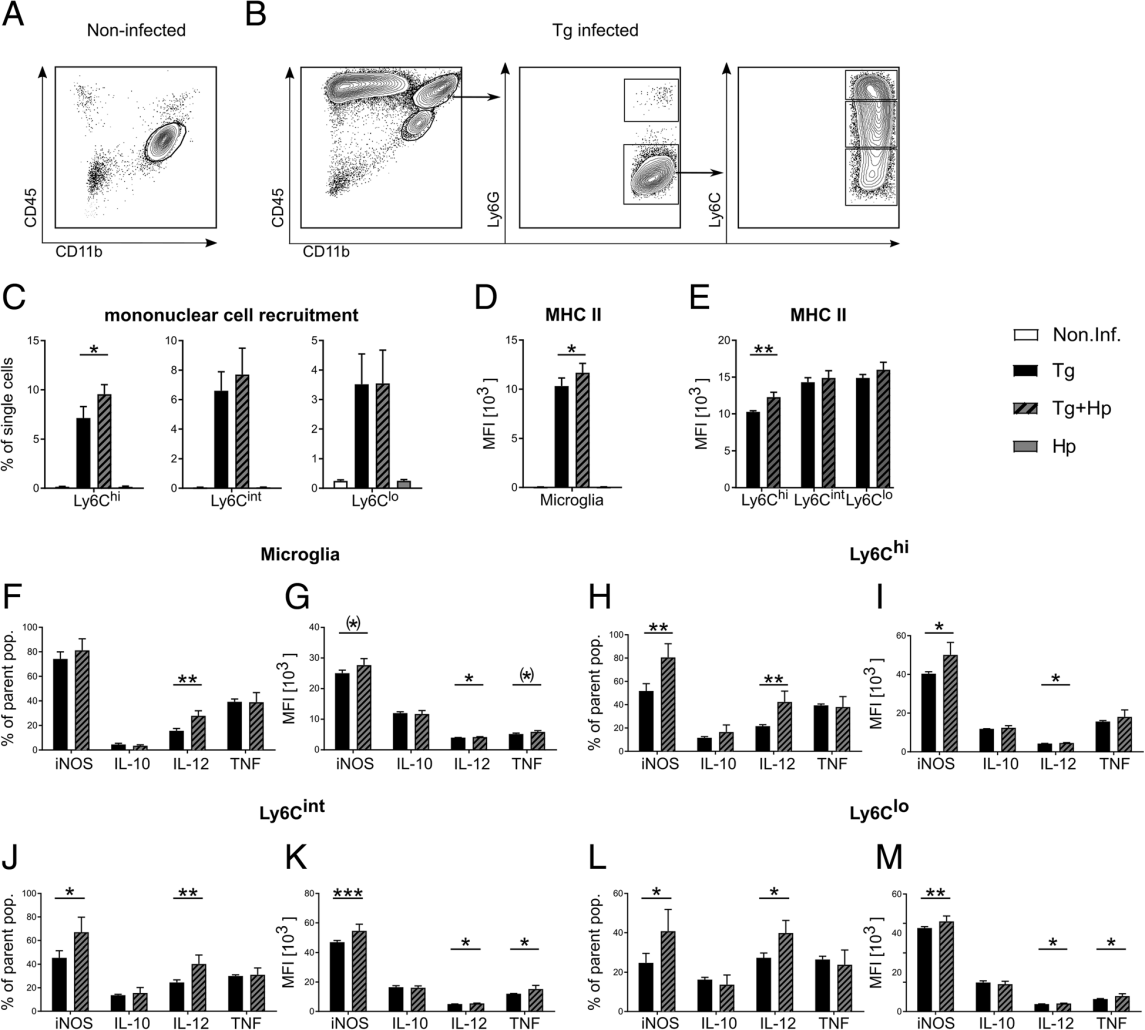
Increased recruitment, MHC II, iNOS, and IL-12 expression in the brain upon *H. polygyrus* co-infection. Immune cells were isolated from the brains non-infected (Non.Inf.), *T. gondii*-infected (Tg), *T. gondii* and *H. polygyrus* co-infected (Tg+Hp), and *H. polygyrus*-infected (Hp) mice and analyzed by flow cytometry. **a**, **b** Representative gating strategy for immune cells. Following viability staining and the basic FSC/SSC gating, single cells were chosen for further characterization. **a** CD11b^+^CD45^int^ cells were identified as microglia. **b** In Tg-infected mice, myeloid cells (CD11b^+^CD45^hi^) were further divided into neutrophils (Ly6G^+^CD11b^+^) and mononuclear cells (Ly6G^−^CD11b^+^). Ly6G^−^Ly6C^+^ mononuclear cells were again divided into three subpopulations (Ly6C^hi^, Ly6C^int^, and Ly6C^lo^). **c** Monocyte recruitment was calculated as a percentage of single cells found in the brain. **d**, **e** Immune cell expression of MHC II was quantified using the MFI of their respective fluorochrome. **f**–**m** Immune cells were further characterized by their intracellular expression of the proteins iNOS, IL-10, IL12, and TNF. Bar graphs represent the percentage of cells positive for the marker of the respective population and the MFI of their respective fluorochrome. Bars represent mean ± SEM

To characterize the cellular composition and uncover the origin of the cytokines responsible for the inflammatory response to the parasites, we analyzed the immune cell subsets in the brain during the infection. The control and subsequent resolution of neuroinflammation requires resident immune cell activation and the persistent recruitment of peripheral immune cells. Accordingly, we characterized resident microglia and recruited immune cell populations. Using hematopoietic cell marker CD45 and myeloid marker CD11b, we distinguished the resident and recruited immune cell populations via fluorescence-assisted cell sorting (FACS), as described previously [23, 72]. In both non-infected mice and mice infected with *H. polygyrus* only, the prevailing immune cell population was the CD45^lo^CD11b^+^ resident microglia, and the infiltration by peripheral CD45^+^ leukocytes was absent (Fig. 3a). In the brains of *T. gondii*-infected and nematode co-infected mice, we observed an infiltration of peripheral CD45^hi^CD11b^−^ lymphocytes and CD45^hi^CD11b^+^ myeloid cells along with a population of activated microglia characterized by an increased expression of CD45 (Fig. 3b).

To further characterize the immune cell recruitment to the brain, we next assessed the abundance of Ly6C monocyte subpopulations as a function of infection status. Recruitment of Ly6C-positive cell populations (Ly6C^hi^, Ly6C^int^, and Ly6C^lo^) was affected by both *T. gondii* (MANOVA with cell populations as dependent variables and pathogens as fixed factor: *F*_3, 11_ = 162.9, Wilks’ *Λ* = 0.022, *p* < 0.001) and, to a lesser extent, *H. polygyrus* (*F*_3, 11_ = 3.82, Wilks’ *Λ* = 0.488, *p* = 0.042), and there was a significant interaction between the two pathogens (*F*_3, 11_ = 3.88, Wilks’ *Λ* = 0.486, *p* = 0.041). While *T. gondii* infection was associated with recruitment of all three cell types (all *F* > 70.99, all *p* < 0.001), *H. polygyrus* and its interaction with *T. gondii* selectively affected the recruitment of Ly6C^hi^ monocytes (all *F* > 10.28, all *p* < 0.007), but not Ly6C^int^ or Ly6C^lo^ cells (all *F* > 0.95, all *p* > 0.348). In direct comparisons, we found a significantly higher proportion of Ly6C^hi^ inflammatory monocytes in the brains of co-infected mice compared to animals solely infected with *T. gondii* (*t*_5.9_ = 3.35, *p* = 0.016; Fig. 3c), whereas no difference was detectable for Ly6C^int^ or Ly6C^lo^ mononuclear cells (all *t* < 1.07, all *p* > 0.320; Fig. 3c).

We next characterized the activation status of microglia and recruited mononuclear cells by assessing their expression status for the class II major histocompatibility complex (MHC II). Because of the very low MHC II expression by microglia in uninfected mice and mice infected with *H. polygyrus* only (Fig. 3d), we restricted statistical analysis to co-infected mice and mice solely infected with *T. gondii*. When testing for MHC II expression across cell populations (microglia, Ly6C^hi^, Ly6C^int^, and Ly6C^lo^), we found a significant increase in MHC II expression in Ly6C^hi^ mononuclear cells (MANOVA with infection status as fixed factor: *F*_4, 3_ = 9.85, Wilks’ *Λ* = 0.071, *p* = 0.045; post hoc *t* test: *t*_3.6_ = 5.82, *p* = 0.006) and a trend-wise increase of MHC II expression in microglia (*t*_5.5_ = 2.12, *p* = 0.041, one-tailed; see Fig. 3d, e) of the co-infected mice, whereas no robust differences were evident for Ly6C^int^ or Ly6C^lo^ cells (all *t* < 1.78, all *p* > 0.065, one-tailed).

Given the importance of the Th1 response in the control of a *T. gondii* infection and the observed reduction of brain parasite burden (see above), we next compared the expression of Th1 response-related genes (IL-12, TNF, iNOS, and IL-10) in recruited mononuclear cells of co-infected animals versus animals infected with *T. gondii* only. When comparing the percentages of cytokine-producing cells (Fig. 3f, h, j, and l) across cell types (microglia, Ly6C^hi^, Ly6C^int^, and Ly6C^lo^), we found a significant modulation of cytokine levels as a function of cell type, infection status, and gene as well as a significant interaction of cell type and infection status and a three-way interaction cell type by gene by infection status (three-way ANOVA for repeated measures with gene and cell type as within-subjects factors and infection status as between-subject factor: all *F* > 12.70, all *p* < 0.009; Greenhouse-Geisser correction applied). These effects could be attributed to co-infected animals showing higher proportions of IL-12-positive cells across all cell types (post hoc two-sample *t* tests: all *t* > 3.29, all *p* < 0.013) and slightly higher proportions of iNOS-expressing Ly6C cells (all *t* > 2.29, all *p* < 0.058, one-tailed), but not microglia (*t*_3.9_ = 1.16, *p* = 0.182, one-tailed). When testing for mean fluorescence intensity (Fig. 3g, i, k, and m), a similar pattern emerged with main effects of cell type, gene, and infection status as well as significant interactions of cell type and infection status, cell type and gene, and gene and infection status (all *F* > 5.37, all *p* < 0.039; Greenhouse-Geisser correction applied), plus a trend-wise three-way interaction (all *F* > 3.17, *p* = 0.063; Greenhouse-Geisser correction applied). These effects could be attributed to increased expression of IL-12 in all cell types of co-infected animals compared to animals infected with *T. gondii* only (post hoc two-sample *t* tests: all *t* > 2.46, all *p* < 0.026, one-tailed) and to all three mononuclear cell subsets (Ly6C^hi^, Ly6C^int^, and Ly6C^lo^) showing a higher expression of iNOS in co-infected animals (all *t* > 2.38, all *p* < 0.027, one-tailed) when compared to only *T. gondii*-only infected mice. Additionally, co-infected animals showed higher expression levels of TNF in Ly6C^int^ and Ly6C^lo^ monocytes (all *t* > 2.61, all *p* < 0.049) and, as a trend, in microglia (*t*_4.9_ = 2.01, *p* = 0.047, one-tailed). IL-10 levels were not affected by *H. polygyrus* in any cell population (all *t* < 1.13, all *p* > 0.157, one-tailed).

**Fig. 4:**
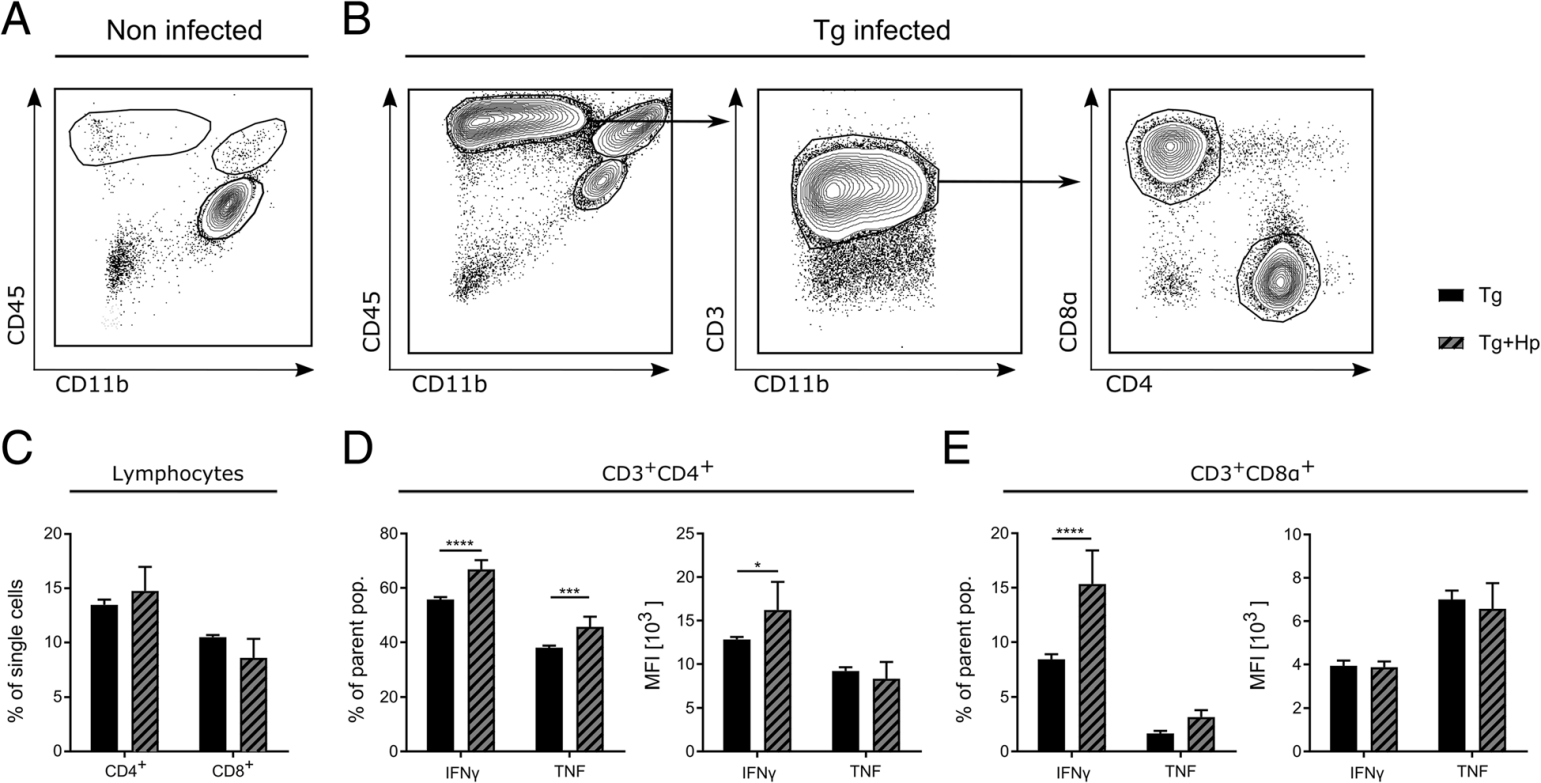
Increased type 1 response in T cells in the brain upon co-infection with *H. polygyrus.* Immune cells were isolated from the brains of *T. gondii-*infected and *T. gondii* and *H. polygyrus* co-infected mice and analyzed by flow cytometry. **a**, **b** Representative gating strategy for lymphocyte populations. Following a viability staining and FSC/SSC gating, single cells were chosen for further characterization. **a** In both non-infected and *H. polygyrus*-infected mice, there was an absence of recruited CD45^+^ leukocytes. **b** In both Tg-infected and Tg and Hp co-infected mice, CD11b^−^CD45^hi^ cells were identified as lymphocytes and then further gated for CD3 expression. CD3^+^ lymphocytes were additionally gated for CD4^+^ and CD8α^+^ T cells. **c** Lymphocyte recruitment was calculated as a percentage of single cells found in the brain. **d**, **e** Intracellular expression of proteins in T cells was characterized by the percentage of cells positive for IFN-γ or TNF and the MFI of the positive cells of each respective marker. Bars represent mean ± SEM

The major role of IL-12 in chronic *T. gondii* infection is sustaining IFN-γ production [69]. Thus, we examined how T cell subsets (the primary IFN-γ producers in chronic infection) are affected by co-infection. CD45^+^CD11b^−^ lymphocytes were further gated for T cells based on CD3 expression and subsequently separated into CD4^+^ and CD8α^+^ populations (Fig. 4a). The recruitment of CD4^+^ or CD8α^+^ T cells to the brain was unchanged in *T. gondii*-infected and co-infected mice (MANOVA with cell type as dependent variable and infection status as fixed factor: *F*_2, 7_ = 2.65, Wilks’ *Λ* = 0.569, *p* = 0.139; Fig. 4c). There was, however, a pronounced increase in the percentage of both CD3^+^CD4^+^ and CD3^+^CD8α^+^ T cells that were positive for IFN-γ and TNF in co-infected compared to *T. gondii*-infected animals (main effect of infection status: *F*_1, 8_ = 43.52, *p* < 0.001; three-way ANOVA for repeated measures with cell type and cytokine as within-subjects factors and infection status as between-subjects factor; post hoc two-sample *t* tests: all *t* > 4.13, all *p* < 0.007; Fig. 4d and e). When comparing mean fluorescence intensities, a three-way ANOVA for repeated measures with cell type and cytokine as within-subjects factors and infection status as between-subjects factor revealed a highly significant three-way interaction of cell type, cytokine, and infection status (*F*_1, 8_ = 15.54, *p* = 0.004), reflecting an increased expression of IFN-γ in CD4^+^ lymphocytes (post hoc two-sample *t* test: *t*_4.3_ = 2.31, *p* = 0.036, one-tailed), while no further significant differences were observed (all *t* < 0.916, all *p* > 0.197, one-tailed). Taken together, these results indicate that *T. gondii* co-infection with an acute *H. polygyrus* infection strongly increases the proportion of IFN-γ-producing T cells in *T. gondii*-infected mice.

### Acute *H. polygyrus* co-infection enhances the *T. gondii* infection-induced decrease of synaptic gene expression

**Fig. 5:**
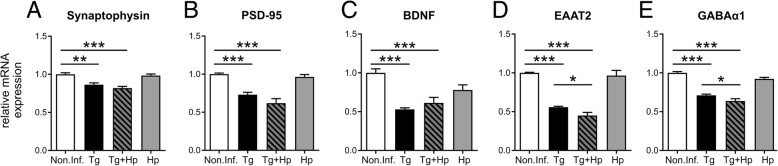
Impairment of synaptic gene expression in the brain upon co-infection with *H. polygyrus.* **a**–**e** qRT-PCR data for relative expression of mRNA in whole brain homogenate from non-infected (Non.Inf.), *T. gondii*-infected (Tg), *T. gondii* and *H. polygyrus* co-infected (Tg+Hp), and *H. polygyrus*-infected (Hp) mice. Relative mRNA levels were normalized to the mean of the non-infected control group (Non.Inf). Bars represent mean ± SEM

Guided by previous observation that *T. gondii*-induced neuroinflammation affects the synaptic protein composition [35], and particularly glutamatergic neurotransmission [34, 73], we next set out to investigate if the enhanced Th1 response influenced the gene expression of synaptic markers in co-infected mice. We focused our analyses on the pre-synaptic vesicle protein synaptophysin (Syp), the post-synaptic scaffolding protein PSD-95 encoded by the *Dlg4* gene, the glutamate transporter EAAT2 encoded by the *Slc1a2* gene, and the α1 subunit of the GABA-A receptor (GABA_A_α1) encoded by the *Gabra1* gene as well as the brain-derived neurotrophic factor (BDNF), given its key role in synaptic plasticity and learning [74, 75]. Expression levels were strongly affected by *T. gondii* (three-way ANOVA for repeated measures with expressed gene as within-subjects factor and *T. gondii* and *H. polygyrus* as between-subjects factors; main effect: *F*_1, 13_ = 193.87, *p* < 0.001; interaction with gene: *F*_1.2, 15.1_ = 166.34, *p* < 0.001) and to a lesser, but significant extent, by *H. polygyrus* (main effect: *F*_1, 13_ = 6.68, *p* = 0.023; interaction with gene: *F*_1.2, 15.1_ = 6.26, *p* = 0.038), whereas no formal interactions between the pathogens were observed (all *F* < 2.00, all *p* > 0.208). All genes investigated were markedly reduced in mice infected with *T. gondii* compared to uninfected mice (post hoc two-sample *t* tests: all *t* > 4.98, all *p* < 0.002; Fig. 5a–e), in line with our previous proteomics study [35]. Co-infection aggravated this pattern, as expression of EAAT2 and GABA_A_α1 was significantly lower in co-infected mice compared to mice infected with *T. gondii* only (EAAT2: *t*_4.3_ = 2.90, *p* = 0.041; GABA_A_α1: *t*_5.1_ = 2.56, *p* = 0.050; Fig. 5d, e). When compared to uninfected mice, mice infected with *H. polygyrus* alone showed reduced expression levels of GABA_A_α1 (*t*_4.4_ = 3.75, *p* = 0.017) and BDNF (*t*_4.3_ = 4.32, *p* = 0.011; Fig. 5c, e). Collectively, these data suggest that an acute nematode co-infection amplifies the decrease of synaptic protein gene expression induced by *T. gondii*, possibly by exerting an additive effect via systemic inflammatory activity.

### Sulfadiazine reduces inflammation and attenuates loss of synaptic gene expression

**Fig. 6:**
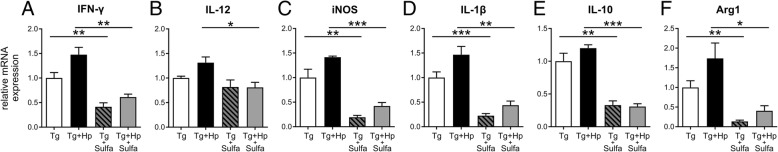
Sulfadiazine treatment reduces the gene expression of pro-inflammatory mediators upon *T. gondii* infection. RNA was isolated from whole brain homogenate of *T. gondii*-infected and co-infected mice with or without sulfadiazine treatment. (**a**–**f**) qRT-PCR data for relative expression of mRNA in whole brain homogenate from *T. gondii*-infected (Tg), *T. gondii* and *H. polygyrus* co-infected (Tg+Hp), *T. gondii*-infected and sulfadiazine-treated (Tg + Sulfa), and *T. gondii* and *H. polygyrus* co-infected and sulfadiazine treated (TgHp + Sulfa) mice. Relative mRNA levels were normalized to the mean of the *T. gondii*-infected group. Bars represent mean±SEM

We next treated mice with sulfadiazine, which targets the fast-replicating tachyzoite stages of infection and as a consequence markedly reduces the strength of the Th1 inflammatory response [76]. To assess if sulfadiazine treatment could indeed reduce *T. gondii*-induced neuroinflammation, we investigated how pro-inflammatory cytokine gene expression was affected. When comparing expression levels of inflammation-related genes in *T. gondii*-infected and co-infected mice treated with sulfadiazine to those in untreated animals, we observed significant effects of *H. polygyrus* infection (main effect: *F*_1, 13_ = 16.94, *p* = 0.001; three-way ANOVA for repeated measures with gene as within-subjects factor and *H. polygyrus* infection and sulfadiazine treatment as between-subjects factors) and treatment (main effect: *F*_1, 13_ = 82.73, *p* < 0.001), but only a trend-wise interaction. Animals infected with *T. gondii* alone showed a treatment-related reduction of gene expression for IFN-γ, iNOS, IL-1β, IL-10, and Arg1 (post hoc two-sample *t* tests: all *t* > 4.61, all *p* < 0.008; Fig. 6a, c–f). When comparing untreated co-infected mice to co-infected mice treated with sulfadiazine, the same genes showed significantly reduced expression, and there was additionally a significant reduction of IL-12 gene expression (all *t* > 3.22, all *p* < 0.036; Fig. 6a–f).

**Fig. 7:**
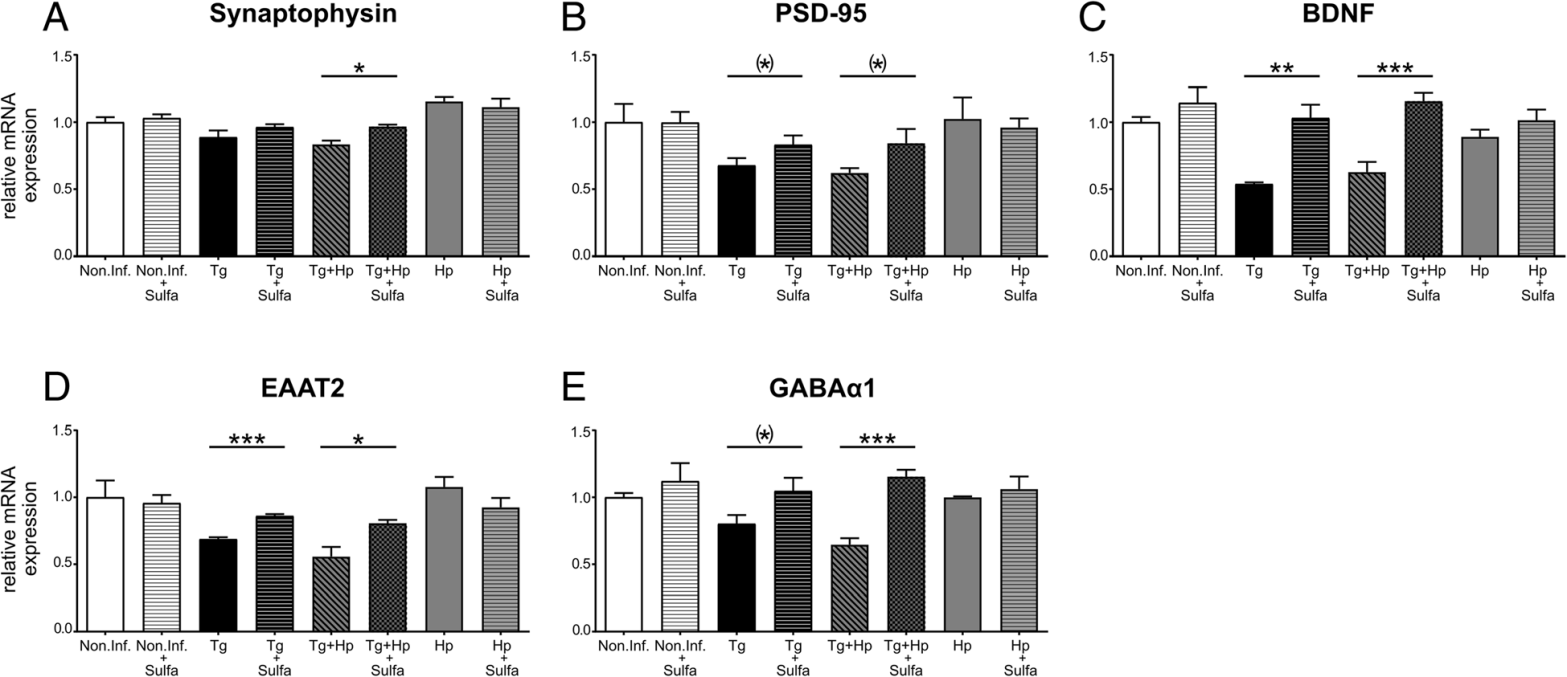
Sulfadiazine treatment prevents *T. gondii* infection-induced decrease of synapse-related gene expression. Non-infected, *T. gondii*-infected, *H. polygyrus*-infected, and co-infected mice were treated with sulfadiazine, and RNA isolated from whole brain homogenate were isolated. **a**–**e** qRT-PCR data for relative expression of mRNA from non-infected (Non.Inf.), non-infected + sulfadiazine treatment (Non.Inf.+sulfa), *T. gondii*-infected (Tg), *T. gondii*-infected + sulfadiazine treatment (Tg+sulfa), *T. gondii* and *H. polygyrus* co-infected (Tg+Hp), *T. gondii* and *H. polygyrus* co-infected + sulfadiazine treatment (Tg+Hp+sulfa), *H. polygyrus*-infected (Hp), and *H. polygyrus*-infected + sulfadiazine treatment (Hp+sulfa) mice. Relative mRNA levels were normalized to the mean of the non-infected control group (Non.Inf.). Bars represent mean±SEM

To assess whether a reduced neuroinflammatory response in sulfadiazine-treated animals would be associated with an attenuation of the synaptic alterations, we next examined the effects of sulfadiazine treatment on the reduction of synaptic marker gene expression. As expected, we observed effects of *T. gondii* infection (four-way ANOVA for repeated measures with gene as within-subjects factor and *T. gondii*, *H. polygyrus*, and sulfadiazine treatment as between-subjects; main effect: *F*_1, 23_ = 55.09, *p* < 0.001; interaction with gene: *F*_1.4, 33.0_ = 30.54, *p* < 0.001) and co-infection (interaction *T. gondii* * *H. polygyrus*: *F*_1, 23_ = 6.14, *p* = 0.021; interaction with gene: *F*_1.4, 33.0_ = 3.97, *p* = 0.041). More importantly, there was a significant interaction between *T. gondii* infection and sulfadiazine treatment (*F*_1, 23_ = 11.70, *p* = 0.002; interaction with gene: *F*_1.4, 33.0_ = 6.68, *p* = 0.008) and a trend-wise three-way interaction (*T. gondii* * *H. polygyrus* * treatment: *F*_1, 23_ = 3.53, *p* = 0.073). Compared to untreated animals, *T. gondii*-infected mice treated with sulfadiazine to infected showed a significantly higher expression of EAAT2 (post hoc two-sample *t* test: *t*_6.0_ = 8.82, *p* < 0.001) and BDNF (*t*_3.1_ = 4.91, *p* = 0.015). In co-infected mice, the attenuation of synaptic gene expression loss by sulfadiazine treatment was even more pronounced with all genes examined except PSD-95 showing significantly higher expression levels in treated animals (all *t* > 3.26, all *p* < 0.035). On the other hand, sulfadiazine treatment did not significantly affect the synaptic gene expression in mice infected with *H. polygyrus* only or in non-infected mice (all *t* < 1.47, all *p* > 0.192), thus excluding unspecific effects of sulfadiazine on gene expression of synaptic proteins (Fig. 7a–e). Taken together, these results suggest that sulfadiazine treatment successfully reduced the strength of the *T. gondii* infection-induced neuroinflammation, thereby attenuating changes to synaptic gene expression.

### IFN-γ neutralization inhibits antiparasitic response and partly recovers synaptic gene expression

**Fig. 8:**
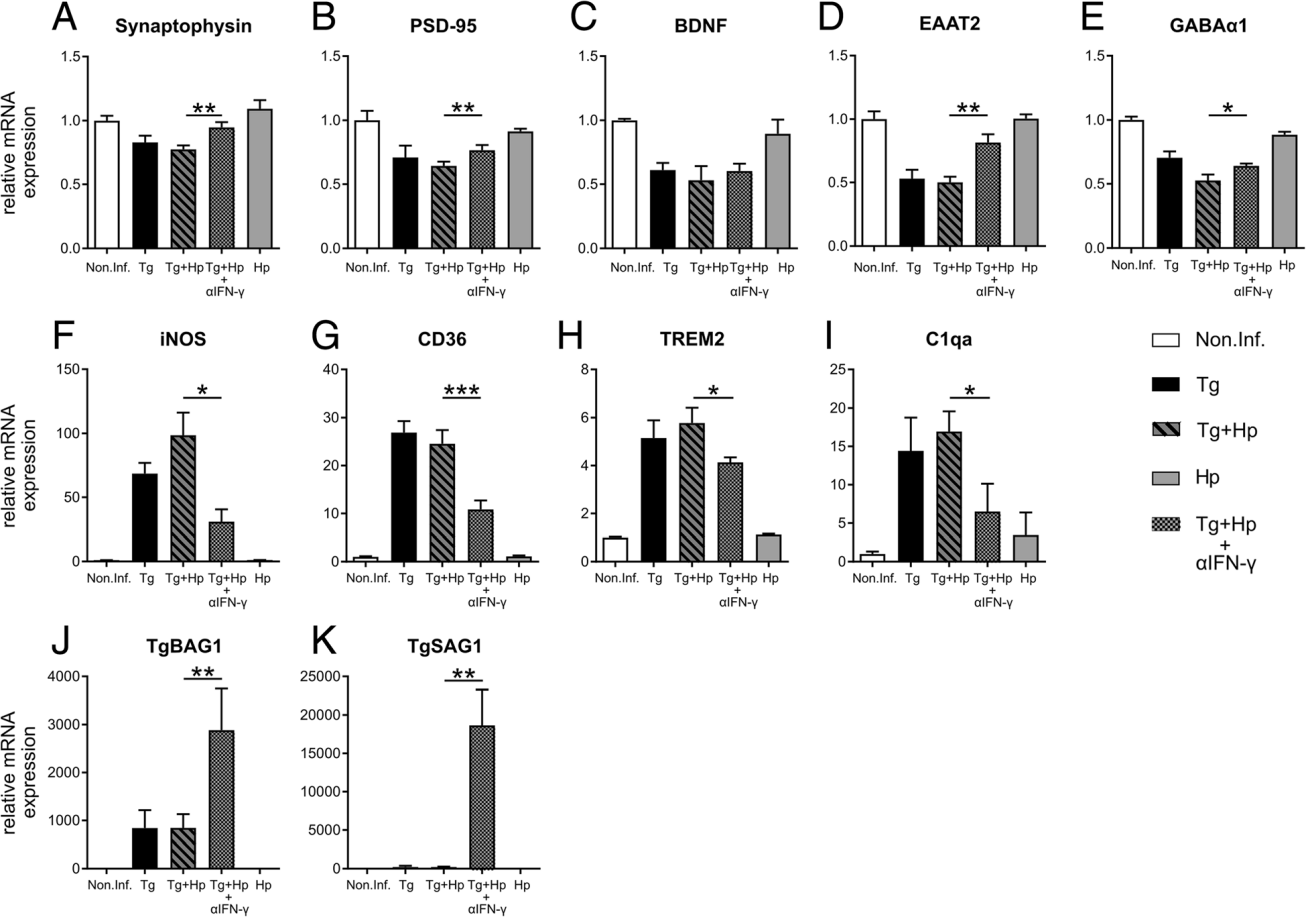
IFN-γ neutralization partially prevents synaptic gene expression, decreases iNOS, CD36, TREM2, C1qa expression and enhances parasite burden in the CNS. Co-infected mice were treated with 0.5 mg of anti-IFN-γ or IgG1 at days 14, 17, 20, 23, and 26 post-infection. **a**–**k** qRT-PCR data for relative expression of mRNA in brain cortex homogenate from non-infected (Non.Inf.), *T. gondii*-infected (Tg), *T. gondii* and *H. polygyrus* co-infected with IgG1 injection (Tg+Hp), *T. gondii* and *H. polygyrus* co-infected with IFN-γ neutralization (αIFN-γ), and *H. polygyrus*-infected (Hp) mice. Relative mRNA levels were normalized to the mean of the non-infected control group (Non.Inf). Bars represent mean ± SEM

Considering the key role of IFN-γ in the antiparasitic response and its previously described effects on GABAergic signaling [6], along with the reduction in EAAT2 and GABA_A_α1 mRNA expression observed here, we hypothesized that neutralizing IFN-γ would rescue the loss of synaptic gene expression described above. When co-infected mice were treated with IFN-γ-neutralizing antibodies, gene expression of synaptic proteins was significantly changed compared to untreated co-infected animals (main effect of αIFN-γ treatment: *F*_1, 8_ = 13.60, *p* = 0.006; interaction treatment by gene: *F*_1.2, 9.4_ = 12.10, *p* = 0.005; two-way ANOVA for repeated measures with gene as within-subjects factor and treatment as between-subjects factor). Specifically, IFN-γ-neutralized animals exhibited significant gene expression level changes for Syp, PSD-95, and EAAT2 (post hoc two-sample *t* tests: all *t* > 2.47, all *p* < 0.040) as well as a trend-wise rescue of GABA_A_α1 expression (*t*_4.2_ = 2.70, *p* = 0.026, one-tailed) (Fig. 8a, b, d, e), whereas BDNF showed no difference in gene transcription as a function of αIFN-γ treatment (Fig. 8c).

We next examined how αIFN-γ treatment affected the parasite burden of the co-infected mice. We found the specific antigens of both tachyzoites and bradyzoites to be significantly increased in IFN-γ-neutralized mice when compared to untreated co-infected animals (*Tgbag1*: *t*_5.0_ = 4.51, *p* = 0.006; *Tgsag1*: *t*_5.4_ = 5.37, *p* = 0.002, Fig. 8j, k). As a reduced parasite load could thus not explain the rescue of synaptic gene expression, we next aimed to elucidate if neurotoxic or phagocytic immune cell activity might be involved in the altered synaptic gene expression. To assess potentially increased cytotoxic activity, we examined expression of iNOS in αIFN-γ-treated compared to untreated animals. Indeed, iNOS expression decreased as a function of IFN-γ antagonization (*t*_4.7_ = 3.34, *p* = 0.022). We next aimed to test whether phagocytic activity might be affected by αIFN-γ treatment and thus examined gene expression of CD36 and TREM2 (markers involved in phagocytosis, parasite killing, scavenging, and synaptic pruning) in treated compared to untreated animals. Both markers were significantly reduced in αIFN-γ-treated compared to untreated co-infected mice (two-way ANOVA for repeated measures with gene as within-subjects factor and αIFN-γ treatment as between-subjects factor: main effect: *F*_1, 8_ = 18.04, *p* = 0.002; interaction: *F*_1, 8_ = 6.43, *p* = 0.035; post hoc two-sample *t* tests: CD36: *t*_7.1_ = 5.08, *p* = 0.001; TREM2: *t*_4.1_ = 3.42, *p* = 0.025) (Fig. 8f–i), suggesting a role for phagocytosis and synaptic pruning in the reduced synaptic gene expression. As phagocytosis is typically promoted by activation of the complement system, we further tested for C1qa expression in treated and untreated mice and observed a small but significant reduction of C1qa mRNA expression upon αIFN-γ treatment (*t*_8.0_ = 2.33, *p* = 0.048).

## Discussion

In this study, we investigated if *T. gondii* infection-induced chronic neuroinflammation can be modulated by a consecutive intestinal infection with *H. polygyrus*. Our findings suggest that an acute nematode infection resulted in enhanced *T. gondii* removal, though at the same time exacerbated the inflammation-related decrease of gene expression of synapse-associated proteins.

Co-infection of *T. gondii*-infected mice with *H. polygyrus* was associated with a significant increase of the Th1-type response, whereas the IL-4 associated Th2 response was comparably suppressed in the brains of co-infected mice. The positive effect of IL-4 in the generation of Th1-mediated immunity in the periphery has been previously acknowledged [77, 78]. The reason for this regulation is that CD8 T cell-derived IL-4 is responsible for maturation of dendritic cells that in turn accelerate Th1 immune responses [79]. Previous studies have shown that *H. polygyrus* infection was able to impede the *T. gondii*-associated Th1 response; however, in those experiments, the nematode was applied prior to *T. gondii* infection [60, 80]. Moreover, we have previously reported that co-infection of mice with *T. gondii* and a nematode did not alter the course of Th1 infection in the gut, but we had used a high dose of *T. gondii* to induce ileitis and mice succumbed in the acute phase of the infection [81]. We have recently reported, using a similar experimental setup, that polarization of peripheral CD4^+^ T cells was disturbed in mice previously infected with *T. gondii* and later co-infected with *H. polygyrus* [82]. Of note, *T. gondii* infection did not prevent *H. polygyrus*-specific priming of T cells, but only the polarization to a Th2 phenotype, as *H. polygyrus*-reactive CD4^+^ T cells upregulated T-bet expression (a Th1 transcription factor that controls IFN-γ expression) and IFN-γ production. The T cells that specifically recognized *H. polygyrus* antigens nevertheless responded in a similar way as Th1 *T. gondii*-specific CD4^+^ T cells [82]*.* This may explain the reduction of parasite burden and increase in nematode fecundity in the co-infected animals. Immune cells responding to *H. polygyrus*, continuously present in the intestine, exhibit Th1 effector functions and further direct circulating immune cells towards a Th1 fate. Our current results align with this hypothesis. We found recruited CD4^+^, CD8^+^, and mononuclear cells with significant increases of the proportion of cells exhibiting Th1 effector function as determined by IFN-γ, iNOS, and IL-12 expression. In line with our results, T cells exhibiting a plastic Th1/Th2 shift have also been shown in a helminth/plasmodium co-infection model [64]. *H. polygyrus*-primed cells adoptively transferred into *Plasmodium chabaudi*-infected animals were converted to IFN-γ-producing cells and subsequently decreased in parasitemia. Future studies should investigate which immune cells mediate the decrease in parasite burden and whether they are *T. gondii* or *H. polygyrus*-specific.

Under homeostatic conditions, distinct cytokines exert a fine-tuned modulatory influence on learning, memory, and synaptic transmission in the CNS. During an infection, increased production of pro-inflammatory cytokines can disrupt the balance of neuro-immune interactions and lead to detrimental effects on neural plasticity and memory as well as neurogenesis and neuronal survival [83]. Pathologically increased levels of IL-1β and TNF can induce degeneration of neurons, alter synaptic scaling, and lead to hyperexcitability, which impairs motor and cognitive performance [7, 8, 84]. Along the same line, nitric oxide (NO) released from activated microglia via iNOS inhibits neuronal respiration that results in glutamate release, the principal excitatory neurotransmitter [85]. NO can also block the presynaptic reuptake of glutamate [86, 87] thereby leading to excitotoxicity and ultimately neurodegeneration. Consistently, in our present setting, co-infected mice exhibited a significant increase of microglia, myeloid cells, and T cells producing iNOS, IL-12, TNF, and IFN-γ, as well as an increase in gene expression of pro-inflammatory proteins IFN-γ, TNF IL-6, IL-1β, and iNOS. Previous studies have convergingly shown that *T. gondii* infection-induced neuroinflammation is associated with markedly reduced expression of key synaptic proteins, including EAAT2, PSD-95, or Shank3 [34, 35], which may lead to increased extracellular glutamate levels and resulting excitotoxicity [73]. Correspondingly, in the present study, both *T. gondii*-infected and co-infected mice displayed a significant decrease in gene expression of synaptic markers synaptophysin, PSD-95, BDNF, EAAT2, and GABA_A_α1. Notably, expression of glutamate transporter EAAT2 and GABA-A receptor subunit GABA_A_α1 were further significantly reduced in co-infected mice compared to mice infected with *T. gondii* only. With EAAT2 being critical for clearance of extracellular glutamate from the synaptic cleft and GABA_A_α1 being a key component of inhibitory GABAergic signaling, their conjoint downregulation is likely to shift the delicate excitation-inhibition balance towards excitation, thereby promoting excitotoxicity (see also [73]). Of note, it is difficult to deduce the loss of synaptic integrity from gene expression alone. For example, Brooks et al. described altered GABAergic signaling in *T. gondii* infection [88]. Specifically, they found that the infection led to a loss of synaptic clustering of the GABA-synthesizing enzyme glutamate decarboxylase 67 (GAD67) in infected animals, resulting in a more diffuse localization throughout the neuropil. Changes in synaptic protein function were not always reflected by altered gene expression. We have previously reported a more detailed record of *T. gondii*-related changes of synaptic biochemistry at the protein level [35], and the present results add to those observations by demonstrating an aggravating effect on synaptic alterations by an acute inflammation induced by the nematode co-infection. There was an exacerbation of neuroinflammation upon co-infection that reduced gene expression of synaptic proteins further compared to animals infected with *T. gondii* only, potentially leaving mice more prone to excitotoxicity and neurodegenerative states.

To test whether the observed changes in neuronal gene expression were induced by the *T. gondii*-induced inflammatory milieu, we treated mice with sulfadiazine, which eliminates extracellular parasites, thus indirectly alleviating inflammatory responses [35]. Remarkably, the resulting reduction of neuroinflammation was associated with an attenuated decrease in gene expression of synaptic-associated proteins, supporting the hypothesis that the inflammatory response is responsible for the neuronal alterations.

Given the increased gene expression of IFN-γ in infected animals and its functions in the immune response to *T. gondii*, we hypothesized that antagonizing IFN-γ signaling would help to clarify the cause of the changes in gene expression of synapse-associated proteins. Consistent with the hypothesis that the specific inflammatory milieu was responsible for reduced synaptic gene expression, neutralizing IFN-γ resulted in a partial rescue of the synaptic gene expression loss. The gene expression levels of synaptic proteins previously shown to be downregulated in *T. gondii*-infected animals, such as EAAT2 and GABA_A_α1 [73, 88], were significantly higher when animals were treated with an antibody directed against IFN-γ. It was recently demonstrated that priming of microglia with IFN-γ induced activation, MHC II upregulation, followed concomitant iNOS production responsible for neuronal network dysfunction involving inhibitory interneurons [89]. In our experiments we also detected IFN-γ associated MHC II, iNOS, and IL-12 upregulation in isolated microglia cells and in recruited monocytes. Moreover, increased expression of EAAT2 and GABA_A_α1, as key components in the excitation/inhibition balance, was associated with reduced IFN-γ and iNOS levels pointing towards a possible mechanism for their regulation.

To further elucidate the underlying molecular mechanisms that might be responsible for the immune response-related neuronal alterations, we focused on cells with phagocytic capacity. Under normal homeostatic conditions, microglia have been shown to engulf neural progenitor cells and synapses. This phagocytic activity is often induced upon release of intracellular molecules (e.g., ATP) or tagging via the complement system [5]. Microglial-mediated phagocytosis of synapses depends on surface receptors recognizing their ligand. Triggering receptor expressed on myeloid cells 2 (TREM2) is a microglial receptor that mediates phagocytosis apoptotic cell debris and developmental synaptic pruning [90]. Similarly, CD36, a scavenger receptor on microglia and infiltrating monocytes, will recognize host and pathogen-derived molecules leading to upregulation of inflammatory molecules (i.e., iNOS and Nox-2) [91]. Both CD36 and TREM2 are understood to be neuroprotective; however, in pathological conditions like neuroinflammation, these have been associated with neurodegenerative diseases such as AD [65, 92, 93]. Therefore, we characterized gene expression of phagocytic markers associated with synaptic pruning such as CD36, TREM2, and C1qa. Our results showed a significant increase in pro-inflammatory cytokine production indicative of prolonged CNS neuro-immune activity. Furthermore, we observed a significant reduction of CD36, TREM2, and C1qa upon IFN-γ neutralization in co-infected mice, suggesting prolonged presence of activated phagocytes and pro-inflammatory cytokines is contributing to the loss of synaptic gene expression. Future studies should dissect the involvement and phagocytic capacity of resident and recruited immune cells that alter synapses. It should be noted that mice in complete lack of IFN-γ rapidly succumb to *T. gondii* infection [28, 94], and we thus reduced IFN-γ levels only partially when applying the neutralizing antibody. Accordingly, the reduction of CD36, TREM2, and C1qa expression was also incomplete with the genes still being expressed, albeit at significantly lower levels. Therefore, we cannot conclusively state whether the partial recovery of synaptic gene expression in *T. gondii*-infected and co-infected mice treated with the IFN-γ antibody was due to inflammatory activity induced by remaining low levels of IFN-γ, due to other proinflammatory cytokines, or due to definite effects of the parasite itself. Thus, subtle alterations of neural function induced by prolonged low-grade inflammation should not be ignored. Even post-pathogen elimination, adverse effects induced by neuroinflammation may manifest as neuronal and behavioral alterations or cognitive impairment [95–99]. Accordingly, it is likely that multiple mechanisms are involved in synaptic gene expression changes; nevertheless, the critical involvement of IFN-γ was revealed.

## Conclusion

Taken together, our data suggest that a neuroinflammatory response to *T. gondii* can be modulated by a strictly enteric helminth co-infection. While consecutive *H. polygyrus* co-infection promoted elimination of *T. gondii*, it adversely affected neural function and synapse-associated gene expression in an IFN-γ-dependent fashion. Helminth immunotherapy has to some extent proven to be successful in chronic inflammatory diseases such as autoimmune disorders or allergies [44, 46, 54, 55]. Regarding potential clinical implications, *T. gondii* seropositive individuals may be at risk for developing neuropsychiatric disorders [18, 100–102]; thus, future research should assess to what extent this risk may be further aggravated by manipulation of the peripheral inflammation [83].

## Additional file

Additional file 1: **Figure S1.** Reduced IL-4 and IL-13 gene expression in the spleen of co-infected mice. (A-B) qRT-PCR data for relative expression of mRNA in the spleen homogenate from non-infected (Non.Inf.), *T. gondii*-infected (Tg), *T. gondii* and *H. polygyrus* co-infected (Tg+Hp), and *H. polygyrus*-infected (Hp) mice. Relative mRNA levels were normalized to the mean of the non-infected control group (Non.Inf). Bars represent mean ± SEM. (PDF 33 kb)

## Data Availability

The datasets used and/or analyzed during the current study are available from the corresponding author on reasonable request.

## Abbreviations

AD: Alzheimer’ disease
ALS: Amyotrophic lateral sclerosis
Arg: Arginase
BDNF: Brain-derived neurotrophic factor
CD: Cluster domain
CNS: Central nervous system
EAAT2: Excitatory amino acid transporter 2
FACS: Fluorescence-activated cell sorting
GABA: Gamma-aminobutyric acid
Hp: *Heligmosomoides polygyrus*
IFN: Interferon
IL: Interleukin
iNOS: Inducible nitric oxide synthase
MHC: Major histocompatibility complex
MS: Multiple sclerosis
PD: Parkinson’s disease
PSD-95: Postsynaptic density protein 95
Tg: *Toxoplasma gondii*
Th1/Th2: Type 1/type 2
TNF: Tumor necrosis factor
TREM2: Triggering receptor expressed on myeloid cells 2
