# Supplementary figures and images for: Neuronal impairment following chronic Toxoplasma gondii infection is aggravated by intestinal nematode challenge in an IFN-γ-dependent manner
Source: J Neuroinflammation. 2019 Jul 29;16:159. doi: 10.1186/s12974-019-1539-8 (PMC6661741; doi:10.1186/s12974-019-1539-8)

# Spleen

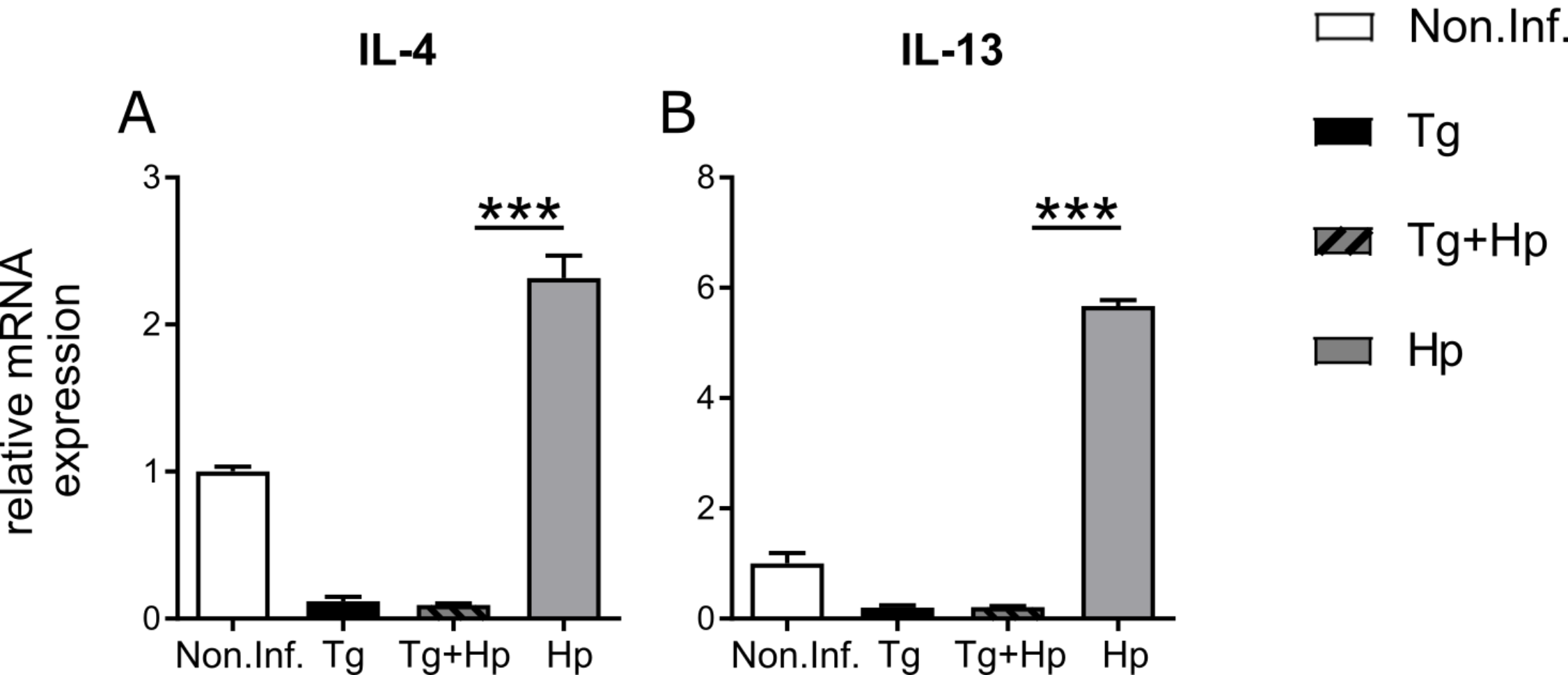

Supplement: Supplementary file 1 — Figure S1. Reduced IL-4 and IL-13 gene expression in the spleen of co-infected mice. (A-B) qRT-PCR data for relative expression of mRNA in the spleen homogenate from non-infected (Non.Inf.), T. gondii-infected (Tg), T. gondii and H. polygyrus co-infected (Tg+Hp), and H. polygyrus-infected (Hp) mice. Relative mRNA levels were normalized to the mean of the non-infected control group (Non.Inf). Bars represent mean ± SEM. (PDF 33 kb) [file 12974_2019_1539_MOESM1_ESM.pdf]
